# Molecular Characterization of HN1304M, a Cat Que Virus Isolated from Midges in China

**DOI:** 10.3390/pathogens11091049

**Published:** 2022-09-15

**Authors:** Ziqian Xu, Lei Cao, Liang Cai, Shihong Fu, Kai Nie, Qikai Yin, Yuxi Cao, Guoping Liu, Yunzhi Liu, Hong Zhang, Lidong Gao, Ying He, Huanyu Wang, Guodong Liang

**Affiliations:** 1 State Key Laboratory of Infectious Disease Prevention and Control, National Institute for Viral Disease Control and Prevention, Chinese Center for Disease Control and Prevention, Beijing 102206, China; 2 Hunan Center for Disease Control and Prevention, Changsha 410005, China; 3 Department of Pest Control, Center for Disease Control and Prevention of Northern Theater Command, Shenyang 110034, China; 4 Foshan Blood Center, Foshan 528000, China

**Keywords:** orthobunyavirus, Manzanilla species complex, Cat Que virus, *Culicoides*, arbovirus

## Abstract

The Cat Que orthobunyavirus has been found in mosquitoes, birds, pigs, and humans, suggesting its wide range of hosts and potential public health implications. During arbovirus surveillance in 2013, the HN1304M virus was isolated from naturally occurring *Culicoides* biting midges in Hunan Province, southern China. The virus was cytopathic to BHK-21 cells and showed stable passage, but was not cytopathic to C6/36 cells. Determination and analysis of the viral genome sequence revealed that HN1304M is an RNA virus with three gene segments, namely, L, M, and S. The nucleotide and amino acid sequence homologies of HN1304M to Cat Que viruses in the Manzanilla species complex were 90.3–99.4%, and 95–100%, respectively, while the homologies to other viruses in this species complex were 74–86.6% and 78.1–96.1%, respectively. A phylogenetic analysis of the viral genes revealed that HN1304M formed an evolutionary branch with other Cat Que viruses isolated from mosquitoes, pigs, birds, and humans, which was completely independent of the other viruses in this complex. The fact that the Cat Que virus was isolated from *Culicoides* suggests that biting midges may participate in the natural circulation of Cat Que viruses.

## 1. Introduction

According to the report of the International Committee on Taxonomy of Viruses (ICTV) published in 2021, the *Bunyavirales* order comprises a group of single-stranded RNA viruses containing 12 viral families, including *Arenaviridae*, *Cruliviridae*, *Fimoviridae*, *Hantaviridae*, *Leishbuviridae*, *Mypoviridae*, *Nairoviridae*, *Peribunyaviridae*, *Phasmaviridae*, *Phenuiviridae*, *Tospoviridae*, and *Wupedeviridae* [1]. *Orthobunyavirus* is the largest genus in the *Peribunyaviridae* family, which contains over 170 viruses [2]. These include several viruses that are pathogenic to humans, including the La Crosse virus, Oropouche virus, and Ngari virus [3,4,5], as well as a variety of viruses that cause animal diseases, including the Aino virus, Akabane virus, Cache Valley virus, and Schmallenberg virus [6]. Serological cross-reactivity studies have demonstrated that the viruses in the *Orthobunyavirus* genus can be divided into 18 serogroups, including Anopheles A, Anopheles B, Bakau, Bunyamwera, Bwamba, group C, Capim, California, Gamboa, Guama, Koongol, Minatitlan, Nyando, Olifanstlei, Patois, Simbu, Tete, and Turlock [7]. Of these, the Simbu serogroup contains 22 viral species that can be divided into seven viral species complexes, namely, Oropouche, Manzanilla, Akabane, Sathuperi, Shamonda, Simbu, and Shuni, based on a molecular evolution analysis of the viral M gene [8].

The Manzanilla species complex includes Manzanilla, Ingwavuma, Mermet, and Cat Que viruses [8], as well as their hosts, including birds, pigs, monkeys, and humans [9,10,11]. Viruses of the Manzanilla species complex have also been isolated from mosquitoes in Vietnam and China [12,13,14]. As viruses of the Manzanilla species complex have been isolated from mosquitoes, birds, and various mammals, it is believed that these viruses follow a virus–mosquito–bird/mammal cycle in nature [15]. However, there are no reports of other blood-sucking arthropods that can serve as vectors for the circulation of viruses of the Manzanilla species complex in nature.

This study reports a virus strain, HN1304M, isolated from midge specimens collected in Hunan Province in southern China, during a study on blood-sucking arthropods, and the virus was found to be cytopathic to mammalian BHK-21cells. Determination and analysis of the whole-genome sequence of HN1304M revealed it to be a Cat Que virus of the Manzanilla species complex. This study is the first to report the isolation of a Cat Que virus from naturally occurring *Culicoides* biting midges. The study also suggested that midges may serve as a transmission vector of Cat Que viruses.

## 2. Results

### 2.1. Specimen Collection and Viral Isolation

A total of 2304 specimens of midges and 7690 mosquito specimens were collected from 7 to 8 August 2013, from three sampling points in Yizhang County of Chenzhou City in Hunan Province of southern China. The longitudinal coordinates of the region are 112°917′ E to 112°971′ E, while the latitudinal coordinates are 25°431′ N to 25°21′ N. According to the morphological characterization, the midges specimens belonged to *Culicoides* genus. *Culex tritaeniorhynchus*, *Anopheles sinensis*, *Armigeres obturbans*, and *Culex quinquefasciatus* accounted for 87% (6653/7690), 11% (839/7690), 2% (168/7690), and 0.4% (30/7690), respectively, of the mosquito specimens collected in this study.

**Figure 1:**
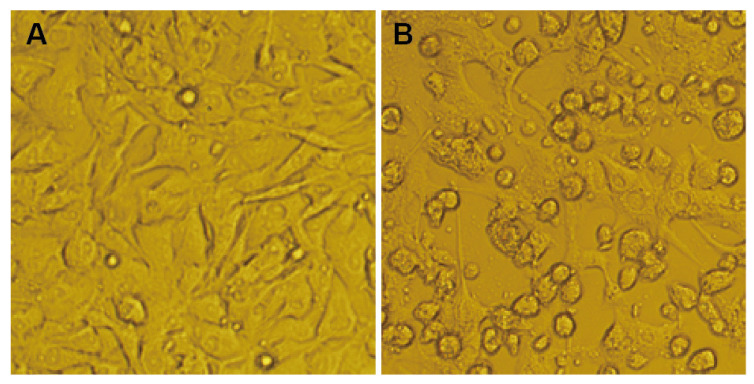
HN1304M causes cytopathic changes in BHK-21 cells. (**A**) Morphology of normal BHK-21 cells; (**B**) morphology of BHK-21 cells after 72 h of infection with HN1304M, showing cell shrinkage and sloughing.

The midge specimens were divided into 20 batches, while the mosquito specimens were divided into 89 batches based on the time of collection, location, and type of arthropod and ground in the laboratory. The ground supernatant of the blood-sucking insects was parallelly inoculated into BHK-21 and C6/36 cell lines, and cytopathic changes were observed on a daily basis. One virus isolate (named HN1304M) was obtained from the *Culicoides* biting midge samples. Following continuous culture and observation, cytopathic changes appeared on the third day of inoculation of the BHK-21 cells (first generation) with the ground supernatant of the midge specimen containing the HN1304M virus, and the lesions reached +++ (75% of the cells exhibited marked cytopathic effect) on the fifth day of inoculation. The supernatant of the diseased cells was continuously passaged, and obvious cytopathic changes were observed in the cells during the second and third passages on the second day of inoculation. The cytopathic changes manifested as cell shrinkage and shedding and reached +++ on the third day of inoculation (Figure 1). It was observed that the HN1304M virus isolate can be stably passaged in BHK-21 cells; however, parallel inoculation of C6/36 cells with the ground supernatant of midges containing the HN1304M virus revealed that the HN1304M virus was not cytopathic to C6/36 cells. The suspension from the fourth passage of the BHK-21 was used as a viral stock and stored at −80 °C. The BHK-21 and C6/36 cells were also inoculated with the ground supernatants of other batches of mosquitoes and midges, and no cytopathic changes were observed.

### 2.2. Molecular Biological Identification of the HN1304M Virus Isolate

#### 2.2.1. Preliminary Identification of HN1304M

In order to identify the HN1304M virus isolate, arbovirus genera-specific primers (Flavivirus, Alphavirus, and Bunyavirus) and virus species-specific primers (Japanese encephalitis virus, Banna virus, Tibet orbivirus, Densovirus, Totivirus, and Oya virus) were used for polymerase chain reaction (PCR)-based detection of the cDNA prepared from the supernatant of the HN1304M virus isolate. The primers used are listed in Supplementary Table S1. The PCR test for the Oya virus was positive, while all of the other PCR tests were negative. Sequencing of the PCR product revealed that the nucleotide sequence of the S gene segment of HN1304M was the most similar (95.0–99.1%) to that of the Cat Que viruses in the Simbu serogroup.

#### 2.2.2. Nucleotide Sequence of the Viral Genome

Based on the genome sequences of the Cat Que viral strains in GenBank, we designed and synthesized amplification primers that cover the full-length gene sequence of the HN1304M virus (Supplementary Table S2). The cDNA prepared from the supernatant from the fourth passage of the BHK-21 cells infected by HN1304M was used as the template, and gene amplification primers were used for amplifying the viral gene sequence and determining its nucleotide sequence. The sequences of the coding regions of the HN1304M viral gene segments were finally obtained. The viral gene sequence information was registered in GenBank (registration numbers: OP129710, OP129711, and OP129712). The genome of the HN1304M virus contains three gene segments, including the L, M, and S segments. The coding region of the L segment has a nucleotide sequence length of 6786 nt and encodes an RNA-dependent RNA polymerase (RdRp) of 2261 aa, while the coding region of the M gene segment has a nucleotide length of 4302 nt and encodes a polyprotein of 1433 aa. The S segment contains two open reading frames (ORFs), and the sequence lengths of the coding regions in this gene segment are 702 nt and 291 nt, which encode a nucleoprotein (NP) of 233 aa and a nonstructural (NS) protein of 96 aa, respectively.

#### 2.2.3. Nucleotide and Amino Acid Sequence Homology of HN1304M Viral Genome

In order to elucidate the molecular characteristics and differences between the HN1304M virus and other related viruses, the viral sequences of representative strains of the Manzanilla species complex, including the Mermet, Ingwavuma, Manzanilla, and Cat Que orthobunyaviruses, and sequences of all viruses related to Cat Que viruses were retrieved from GenBank (Supplementary Table S3). The nucleotide and amino acid sequence homology of HN1304M with the aforementioned viruses was determined using the online BLASTn and BLASTp alignment tools, respectively (https://blast.ncbi.nlm.nih.gov/Blast.cgi) (accessed on 1 August 2022). The results demonstrated that the nucleotide and amino acid sequences of the three gene segments of the HN1304M virus were similar to those of six strains of Cat Que viruses, including the original VN04-2108, GD18234, SC0806, JM1, NIV86209, and DHL10M107 isolates. The nucleotide and amino acid similarities between HN1304M and these six viral isolates were 90.3–99.4% and 95.0–100%, respectively (Table 1). The nucleotide sequence similarity between the HN1304M virus and three other viruses in the Manzanilla virus species complex, including the Manzanilla (TRVL3586), Ingwavuma (SA An 4165), and Mermet (AV 782) orthobunyaviruses, was 74.0–86.6%, while the amino acid sequence similarity was 78.1–96.1%. The similarity between the nucleotide sequences of the HN1304M virus and four other representative members of Clade A viruses in the Simbu serogroup, namely, the Buttonwillow virus (BFS 5002), Facey’s Paddock virus (Aus Ch 16129), Utinga virus (Be An 84785), and a reference strain of the Oropouche virus, was lower at 66.4–75.4%, while the amino acid sequence similarity was 51.5–80.6% (Table 1). The results also demonstrated that the amino acid sequence homology between the HN1304M virus and the original Cat Que viral isolate (VN04-2108) was 100%. These results suggested that the HN1304M virus isolated from *Culicoides* biting midges in this study is a Cat Que virus of the Manzanilla virus species complex.

### 2.3. Phylogenetic Analysis of the HN1304M Virus

**Figure 2:**
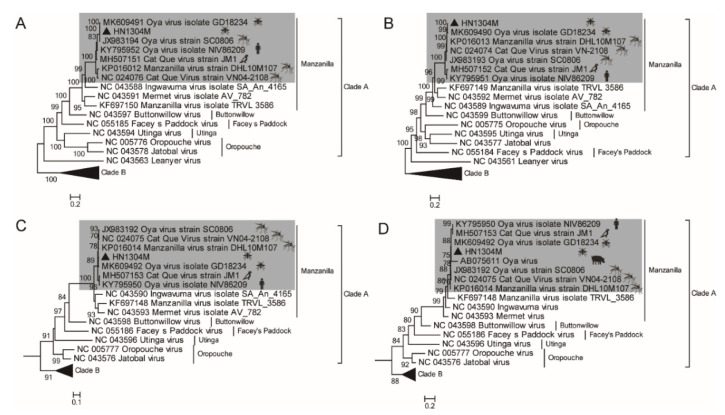
The phylogenetic relationship between the HN1304M virus and other viruses of the Orthobunyavirus genus in the Simbu serogroup was analyzed using the maximum likelihood method. The phylogenetic tree constructed by aligning the nucleotide sequences of the L gene (**A**), M gene (**B**), and S gene (**C**) and a partial sequence of the S gene (365 nt) (**D**). The HN1304M strain is marked with black triangles. The Cat Que virus clade is shaded in gray, where the host origin of the virus strains is indicated by the following symbols:
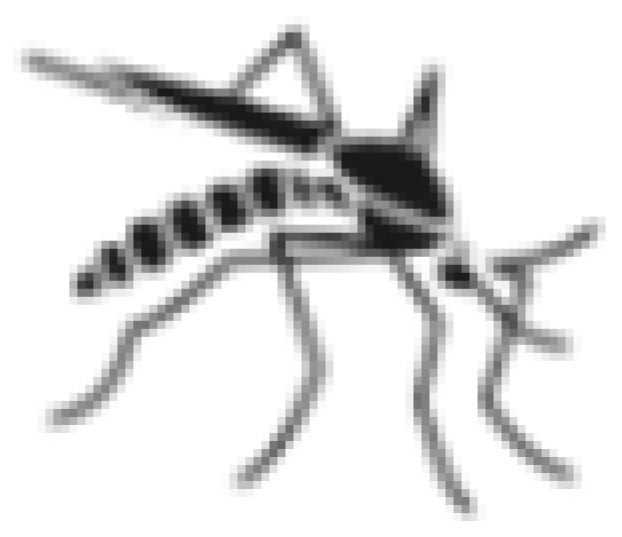
 represents mosquitoes; 
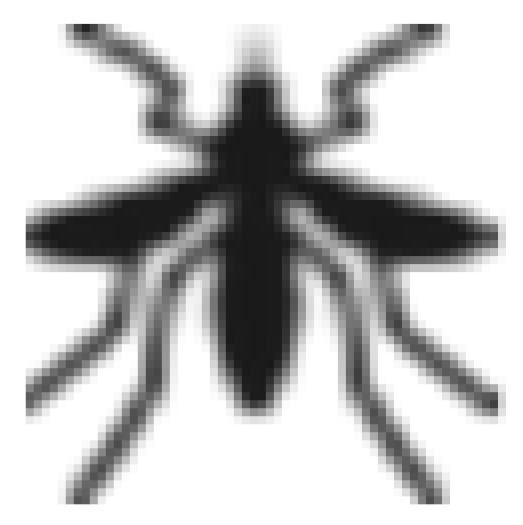
 represents midges;
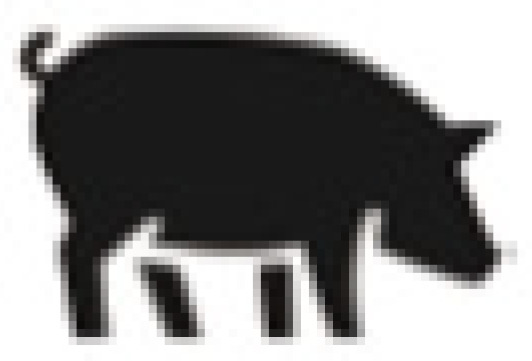
 represents pigs;
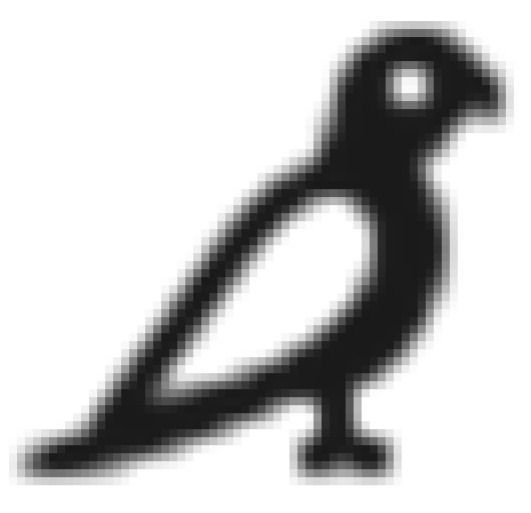
 represents birds; and 
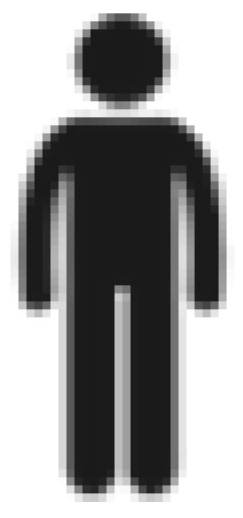
 represents humans.

In order to elucidate the molecular genetic evolutionary characteristics of the HN1304M virus isolated from the midge specimens, this study established a nucleotide sequence dataset of genes from representative strains of Clade A and Clade B viruses of the Simbu serogroup (Supplementary Table S3). Phylogenetic analyses demonstrated that the Clade A viruses in the Simbu serogroup can be divided into five evolutionary lineages, including the Manzanilla, Buttonwillow, Facey’s Paddock, Utinga, and Orpouche lineages. The Manzanilla lineage further contained four viruses, namely, the original VN04-2108 isolate of the Cat Que virus, the Manzanilla orthobunyavirus virus (TRVL3586), the Ingwavuma orthobunyavirus virus (SA An 4165), and Mermet orthobunyavirus (AV 782). Seven viral isolates, including the HN1304M virus, comprised an evolutionary branch together with the original VN04-2108 isolate of the Cat Que virus, suggesting that these viruses belong to the Cat Que virus in the Manzanilla lineage (Figure 2A–C).

During the Nipah virus outbreak in Malaysia in 1999, an orthobunyavirus (Oya virus) was isolated from ailing pigs [10]. Only a 365 nt partial sequence of the S gene segment of the virus is available in GenBank (accession number: JX983192). The 365 nt sequence of JX983192 was aligned with the sequences of the S genes of all corresponding viruses and phylogenetically analyzed in this study. The results demonstrated that the Oya virus that was isolated from ailing pigs in Malaysia in 1999, belonged to the branch of Cat Que viruses of the Manzanilla lineage and was closely related to the HN1304M virus isolated from the *Culicoides* biting midges in this study (Figure 2D). This suggests a potential relationship between the HN1304M virus isolated from midges and the Oya virus (JX983192) isolated from pigs during the Nipah virus outbreak in 1999.

## 3. Discussion

In this study, the HN1304M virus, which can cause mammalian cytopathies, was isolated from naturally occurring *Culicoides* biting midges collected from Hunan Province in southern China. The results of whole-genome molecular biology and phylogenetic analyses revealed that the HN1304M virus isolated from midges was a member of the Cat Que viruses in the Manzanilla species complex of the Simbu serogroup. Cat Que viruses have been previously isolated from mosquitoes [12,13,14], birds, humans [9,15], and pigs [10]; however, this study is the first to report the isolation of a Cat Que virus from *Culicoides* biting midges.

As aforementioned, Cat Que viruses have been isolated from mosquito samples in Vietnam [12], mosquito isolates in the Yunnan Province in China [13], mosquito isolates in Sichuan Province in China [14], birds (myna) and human patient specimens in India [9,15], midges (the HN1304M virus isolated in this study), and ailing pigs during the Nipah virus outbreak in Malaysia in 1999 [10]. These findings demonstrate that Cat Que viruses can be transmitted by mosquitoes and midges, and can infect pigs, poultry (birds), and humans (Figure 2). Seroepidemiological analyses of Cat Que viruses have revealed positive IgM and IgG antibodies in serum samples collected from pigs in the Sichuan area of China, where the Cat Que virus (SC0806) was isolated. The positive rates of IgM and IgG antibodies were 21.98% (20/91) and 60.43% (55/91), respectively, while the positive rate of both antibodies was 7.53% (7/91). The findings demonstrated that infection with Cat Que viruses is common in locally raised pigs. Further analysis revealed that the positive rate of viral IgM antibodies was highest in locally raised piglets younger than 4 months of age, and the positive rate gradually decreased with increasing age, while the positive rate of viral IgG antibodies gradually increased with age, reaching 100% at 8 months of age [14]. The isolation of Cat Que viruses from ailing pigs, mosquitoes, and midges [10,12,13,14], and the high positive rate of Cat Que viral IgM and IgG antibodies detected in the serum of domestic pigs [14] indicate that pigs are an important mammalian host for Cat Que viruses, while mosquitoes and midges serve as arthropod vectors of the virus. Therefore, the Cat Que virus, or even the Manzanilla species complex, could be transmitted following a virus–mosquito/midges–mammal (bird/pig) cycle in nature.

## 4. Materials and Methods

### 4.1. Specimen Collection

Specimens of mosquitoes were collected between 7 and 8 August 2013, from three sampling sites in Yizhang County of Chenzhou City in Hunan Province. The mosquitoes and midges were collected at night using mosquito lure lamps (Kung Fu Xiaoshuai, 12 V, 300 mA; Wuhan Lucky Star Environmental Protection Technology, Hubei, China). The specimens were collected from 16:30 h to 6:30 h the following day. Following a morphology-based classification, the specimens of mosquitos and midges were preserved in separate cryopreservation tubes based on the collection point and morphological classification, stored in liquid nitrogen, and transported to the laboratory on dry ice [14].

### 4.2. Cell Culture Experiments

BHK-21 cells were cultured in minimum essential medium (MEM) supplemented with 10% fetal bovine serum (Gibco), 1% penicillin-streptomycin (100 U/mL), 1% glutamine (30 g/L), and 1% sodium bicarbonate at 37 °C in a 5% CO2 incubator. The C6/36 cell line was cultured in RPMI 1640 medium (Invitrogen) supplemented with 10% fetal bovine serum (Gibco) and 1% penicillin-streptomycin (100 U/mL) in an incubator at 28 °C [16].

### 4.3. Virus Isolation

The samples were washed twice with grinding fluid (MEM supplemented with 5% penicillin-streptomycin (100 U/mL), 1% glutamine (30 g/L), and 1% sodium bicarbonate), following which 1.5 mL of grinding liquid was added, and the samples were ground for 3 min with a tissue grinder (Retsch Tissue Lyser, QIAGEN, Hilden, Germany) at a frequency of 25 times/s. After grinding, the sample was centrifuged at 12,000× *g* for 30 min at 4 °C. The C6/36 and BHK-21 cells were then separately inoculated with 100 μL of the supernatant of the grinding fluid used for culture, and cytopathy was observed on a daily basis [17].

### 4.4. Identification of Virus

Viral RNA was extracted from 140 μL of the cell culture supernatant of BHK-21 cells using an QIAamp Viral RNA Mini Kit (Qiagen), and the cDNA library was prepared using a Ready-To-Go kit (GE Healthcare, Little Chalfont, Buckinghamshire, UK). Common arbovirus primers, including Flavivirus, Alphavirus, and Bunyavirus primers, and species-specific primers for Japanese encephalitis virus, Banna virus, Tibetan orbivirus, Denso virus, Totivirus, and Oya virus (Supplementary Table S1) were used for PCR [18]. The viral sequence was confirmed by designing overlapping PCR primers based on the sequences of the Cat Que viral strains (Supplementary Table S2).

### 4.5. Sequence and Phylogenetic Analysis

The sequences of all viruses in the Manzanilla species complex and other representative viruses of the Simbu serogroup (Supplementary Table S2) were retrieved for sequence and phylogenetic analyses. The sequences were analyzed using the ORF finder tool. The retrieved nucleotide and amino acid sequences of the HN1304M and other representative viruses were aligned using the BLASTp and BLASTn tools of NCBI, respectively. A sequence alignment was performed using the Mafft software (7.450-win), and the sequences that were poorly aligned at the 5′ and 3′ ends were manually removed. The sequences were pruned using the TrimAL software. The phylogenetic tree was constructed with the PhyML-3.1 software using the maximum likelihood method and the SPR structure optimization algorithm [19].

## 5. Conclusions

Cat Que viruses have been previously isolated from mosquitoes in Yunnan [13] and Sichuan [14], as well as from *Culicoides* biting midges in Hunan Province in China, in the present study. These three provinces are located in southern China and are geographically adjacent to each other. These regions have a high annual average temperature, abundant rainfall, and diverse biological species, which are suitable for the breeding of various blood-sucking insects and arboviruses. A variety of arboviruses transmitted by mosquitoes (including the Japanese encephalitis virus and Dengue virus, among others) and midges (including the Yunnan orbivirus and Tibet orbivirus, among others) have been previously isolated from blood-sucking insects in these regions [20]. The Japanese encephalitis virus, Tibet orbivirus, and Akabane virus were isolated in 2010–2011 during an investigation of mosquito-borne viruses in Hunan Province [21]. In this study, a Cat Que virus was isolated from specimens of *Culicoides* biting midges collected in Hunan Province. Cat Que viruses were isolated from mosquitoes during an investigation of arboviruses associated with acute encephalitis in children in Vietnam [12], and were also isolated from pigs [10] and humans [9,15]. Therefore, the detection of Cat Que virus infections in animals, and the timely detection of this virus in human subjects with fever or viral encephalitis, is crucial for the implementation of viral control measures.

## Figures and Tables

**Table 1:** Nucleotide and amino acid sequence identity (%) between the HN1304M virus and representative members of Clade A viruses in the Simbu serogroup of the *Orthobunyavirus* genus.

Lineage	Species	Strains (Host)	L Gene		M Gene		S Gene		
			nt	aa	nt	aa	nt	aa (NP)	aa (NSs)
Manzanilla	*Cat Que orthobunyavirus*	GD18234 (midges)	99.0	99.7	98.4	99.7	99.1	99.6	99.0
		SC0806 (mosquito)	99.4	99.8	96.3	98.9	98.5	100	100
		VN04-2108 (*Culex* sp.)	92.9	99.1	96.9	98.9	98.4	100	100
		JM1 (*Acridotheres fuscus*)	93.1	99.1	90.3	96.2	95.0	99.6	99.0
		NIV86209 (*Homo sapiens*)	95.3	99.2	91.9	97.1	96.8	100	99.0
		DHL10M107 (*C. tritaeniorhynchus*)	92.4	98.9	97.4	99.2	98.9	100	99.0
	*Manzanilla orthobunyavirus*	TRVL3586 (*Alouatta seniculus*)	77.8	89.6	74.0	78.1	83.9	96.1	88.5
	*Ingwavuma orthobunyavirus*	SA An 4165 (*Hyphanturgus ocularis*)	78.3	90.5	74.0	79.4	84.3	92.7	88.5
	*Mermet orthobunyavirus*	AV 782 (*Progne subis*)	77.8	90.7	74.0	80.4	86.6	96.1	86.3
Buttonwillow	*Button willow orthobunyavirus*	BFS 5002 (*Culicoides* sp.)	73.2	80.6	67.4	65.7	75.3	74.7	66.7
Facey’s Paddock	*Facey’s Paddock orthobunyavirus*	Aus Ch 16129 (mosquito)	71.0	75.9	67.9	52.2	69.8	72.8	57.9
Utinga	*Utinga orthobunyavirus*	Be An 84785 (*Bradypus tridactylus*)	69.0	76.1	66.4	54.1	66.6	73.7	NA
Oropouche	*Oropouche orthobunyavirus*	BeAn19991 (*Bradypus tridactylus*)	68.2	69.1	69.9	51.5	72.9	75.4	56.0

NA, not available.

## Data Availability

Sequences were submitted to GenBank with accession numbers OP129710, OP129711, and OP129712.

## Supplementary Materials

The following supporting information can be downloaded at: https://www.mdpi.com/article/10.3390/pathogens11091049/s1, Table S1: PCR primers used for the preliminary identification of the HN1304M virus; Table S2: primers used for amplifying the whole-genome sequence of HN1304M; Table S3: viral strains used in phylogenetic analysis of the isolated HN1304M virus in this study.
