# Supplementary material for: Molecular Characterization of HN1304M, a Cat Que Virus Isolated from Midges in China
Source: Pathogens. 2022 Sep 15;11(9):1049. doi: 10.3390/pathogens11091049 (PMC9501373; doi:10.3390/pathogens11091049)
Supplement: Supplementary file 1 [file pathogens-11-01049-s001.zip › pathogens-1896502-supplementary.pdf]

Supplementary Table S1. PCR primers used for the preliminary identification of the HN1304M virus.

Genus or species	Name of primer	Amplified segment	Sequence of primers (5′-3′)	Length of amplified region (bp)
Flavivirus	FU1	NS5	TAC AAC ATG ATG GGA AAG AGA GAG AA	266
	cFD2		GTG TCC CAG CCG GCG GTG TCA TCA GC	
Alphavirus	M2W(+)	NSP1	YAG AGC DTT TTC GCA YST RGC HW	434
	cM3W		ACA TRA ANK GNG TNG TRT CRA ANC CDA YCC	
	M2W2(+)		TGY CCN VTG MDN WSY VCN GAR GAY CC	310
Bunyavirus	BUP	S	ATG ACT GAG TTG GAG TTT GAT GTC GC	251
	BDW		TGT TCC TGT TGC CAG GAA AAT	
Banna virus	BAV-12-854-S	12S	AAA TTG ATA GYG YTT GCG TAA GAC	850
	BAV-12-B2-R		GTT CTA AAT TGG ATA CGG CGT GC	
Tibet orbivirus	6-4-2F	4S	CGA CAG ACC AAA AGA TAT	848
	6-4-2R		TCA ACA CGT AAT CCA ATA	
Japanese encephalitis virus	JE-955F	E	TGY TGG TCG CTC CGG CTT A	1581
	JE-2536R		AAG ATG CCA CTT CCA CAY CTC	
Densovirus	DNV-F	NS1	AAC CGT TGG TGA CCT CTA CCC AC	1105
	DNV-R		GAT TTT CCC ATT GCT GTG CCG TTG	
Totivirus	Toti2-947F		GCA ATG GAG AAC AAA TTC ACG	825
	Toti2-1771R		CTG GTG TGC TTG TTT CTG TTT C	
Oya virus	Oya-1F	S	AGT AGT GTA CTC CAC AAT TCA AAA	966
	Oya-966R		AGT AGT GTG CTC CCA ATT CAA AGA	

Supplementary Table S2. Primers used for amplifying the whole genome sequence of HN1304M.

Primer	Gene segment	Sequence (5′-3′)	Length of amplified region (nt)
L1F	L	AGTAGTGTACCCCTAGGTTACAACATAC	995
L994R		TCTGAGTTGGGGGACCAGAT	
L890F	L	GGGATGGGCAGAAATGGTTG	806
L1695R		TGTCTGAACTCGGCATCACAA	
L1507F	L	GTGCAAGTGGAAAAATGTGGGA	749
L2255R		AACTTTGCCGGGAAACCAGA	
L1758F	L	GTTGCGCACCTTGGTTCATT	1417
L3174R		GGCCAAGGTCATCTAGGCTG	
L2897F	L	GGGTCAGAAGACGGCTAAAG	1386
L4282R		CCCATCCCATCATCAGTGGT	
L3894F	L	TGCCCGGCATCATTAGCAT	1427
L5320R		TGAACCTCCAAATCTGGCGAA	
L4858F	L	GCAGGATGCCAGAAATGAGG	1344
L6201R		GGGACGCTTGAGCCATTGTA	
L6060F	L	GCCACAGTTAGGAAGGCACA	869
L6928R		AGTAGTGTGCCCCTAGGAACATTAATATAC	
M1F	M	AGTAGTGTACTACCACATACAACAAAC	1326
M1326R		CCACAGATACATGTCCCGCA	
M984F	M	CCTGGTCATTCATCTCTGCCA	1493
M2476R		ATCCGCATCTAGCCTAGGGA	
M2283F	M	TTTGCAAAGAGCCCCACTCTA	1490
M3772R		CCCAAGTGCAATCCGGAAGT	
M3455F	M	CCAGCTCTTATGGGCTTGAGG	1027
M4481R		AGTAGTGTGCTACCACGTACAAACATTC	
S1F	S	AGTAGTGTACTCCACAATTCAAAAAC	657
657R		ATGCTGCCCTAATTGCCACT	
S396F	S	GTCCGTGAGGCAGTTATCAT	589
S984R		AGTAGTGTGCTCCCAATTC	

Supplementary Table S3. Viral strains used in phylogenetic analysis of the isolated HN1304M virus in this study.

Species complex	Species	Name of strains	Year of isolation	Isolated from	Host	GenBank accession number		
						S segment	M segment	L segment
Manzanilla		GD18234	2018	China	Mite	MK609492	MK609490	MK609491
		SC0806	2008	China	Mosquito	JX983192	JX983193	JX983194
		VN04-2108	2004	Vietnam	*Culex* sp.	NC024075	NC024074	NC024076
	Cat Que orthobunyavirus	JM1	1961	India	*Acridotheres fuscus*	MH507153	MH507152	MH507151
		NIV86209	1986	India	*Homo sapiens*	KY795950	KY795951	KY795952
		DHL10M107	2010	China	*Culex tritaeniorhynchus*	KP016014	KP016013	KP016012
		NA	2000	Malaysia	Pig	AB075611	NA	NA
	Manzanilla orthobunyavirus	TRVL3586	1954	Trinidad and Tobago: Trinidad	*Alouatta seniculus*	KF697148	KF697149	KF697150
	Ingwavuma orthobunyavirus	SA An 4165	1959	South Africa	*Hyphanturgus ocularis*	NC043590	NC043589	NC043588
	Mermet orthobunyavirus	AV 782	1964	USA	*Progne subis*	NC043593	NC043592	NC043591

	Button willow orthobunyavirus	BFS 5002	1964	USA	*Culicoides* sp.	NC043598	NC043599	NC043597
	Facey's Paddock orthobunyavirus	Aus Ch 16129	1974	Australia	Mosquito	NC055186	NC055184	NC055185
Oropouche	*Utinga orthobunyavirus*	Be An 84785	1965	Brazil	*Bradypus tridactylus*	NC043596	NC043595	NC043594
	Oropouche orthobunyavirus	BeAn19991	NA	Brazil	*Bradypus tridactylus*	NC005777	NC005775	NC005776
Simbu	*Simbu orthobunyavirus*	SA Ar 53	1955	South Africa	*Aedes circumluteolus*	NC018477	NC018478	NC018476
Shuni	*Shuni orthobunyavirus*	SAE1809	2009	South Africa	Horse	NC043698	NC043697	NC043699
Akabane	*Akabane orthobunyavirus*	OBE-1	1974	Japan	Bovine animals	NC009896	NC009895	NC009894
Shamonda	*Shamonda orthobunyavirus*	Ib An 5550	1965	Nigeria	Cattle	NC018464	NC018467	NC018463

Sathuperi	*Sathuperi orthobunyavirus*	NA	NA	NA	NA	NC018462	NC018466	NC018461

NA，not available.
